# Unified Classification of Bacterial Colonies on Different Agar Media Based on Hyperspectral Imaging and Machine Learning

**DOI:** 10.3390/molecules25081797

**Published:** 2020-04-14

**Authors:** Peng Gu, Yao-Ze Feng, Le Zhu, Li-Qin Kong, Xiu-ling Zhang, Sheng Zhang, Shao-Wen Li, Gui-Feng Jia

**Affiliations:** 1 Department of Mechatronics Engineering, College of Engineering, Huazhong Agricultural University, Wuhan 430070, China; gupenghzau@163.com (P.G.); zhu_le99@163.com (L.Z.); kongliqinhzau@163.com (L.-Q.K.); zs825587003@163.com (S.Z.); guifeng@mail.hzau.edu.cn (G.-F.J.); 2 Key Laboratory of Agricultural Equipment in Mid-Lower Yangtze River, Ministry of Agriculture and Rural Affairs, Wuhan 430070, China; 3 Department of Preventive Veterinary Medicine, College of Veterinary Medicine, Huazhong Agricultural University, Wuhan 430070, China; ZXL20171207@163.com (X.-l.Z.); lishaowen@mail.hzau.edu.cn (S.-W.L.)

**Keywords:** bacterial pathogens, Visible-Near-infrared hyperspectral imaging, grasshopper optimization algorithm, support vector machine, variable selection, optimization, bacterial contamination

## Abstract

A universal method by considering different types of culture media can enable convenient classification of bacterial species. The study combined hyperspectral technology and versatile chemometric algorithms to achieve the rapid and non-destructive classification of three kinds of bacterial colonies (*Escherichia coli*, *Staphylococcus aureus* and *Salmonella*) cultured on three kinds of agar media (Luria–Bertani agar (LA), plate count agar (PA) and tryptone soy agar (TSA)). Based on the extracted spectral data, partial least squares discriminant analysis (PLS-DA) and support vector machine (SVM) were employed to established classification models. The parameters of SVM models were optimized by comparing genetic algorithm (GA), particle swarm optimization (PSO) and grasshopper optimization algorithm (GOA). The best classification model was GOA-SVM, where the overall correct classification rates (OCCRs) for calibration and prediction of the full-wavelength GOA-SVM model were 99.45% and 98.82%, respectively, and the Kappa coefficient for prediction was 0.98. For further investigation, the CARS, SPA and GA wavelength selection methods were used to establish GOA-SVM simplified model, where CARS-GOA-SVM was optimal in model accuracy and stability with the corresponding OCCRs for calibration and prediction and the Kappa coefficients of 99.45%, 98.73% and 0.98, respectively. The above results demonstrated that it was feasible to classify bacterial colonies on different agar media and the unified model provided a continent and accurate way for bacterial classification.

## 1. Introduction

Foodborne pathogens are among the chief culprits of foodborne diseases, which can seriously threaten the life of human beings [1,2,3]. The detection of foodborne pathogens is important for controlling foodborne diseases and many countries have initiated foodborne pathogen surveillance programs to improve food safety and to prevent outbreaks of foodborne diseases [4]. The existing food-borne pathogen detection methods mainly include traditional plate culture detection methods, immunological-based detection methods (including fluorescent antibody detection method [5] and enzyme-linked immunosorbent assays [6], etc.), molecular biology-based detection methods (including polymerase chain reaction [7] and biological gene chip methods [8], etc.). However, most of the current detection methods are still facing challenges of destructive measurement, high instrumental costs, excessive labor work and extended detection time. Therefore, it is of great significance to develop a rapid, non-destructive and efficient method for classifying foodborne pathogens.

As a fast and non-destructive detection technology, hyperspectral technology has been widely used to analyze internal physical structure and biochemical composition information of biological samples [9]. Its great potential application in the rapid identification of bacterial colonies has been well demonstrated [10]. Giovanni Turra et al. [11] achieved the discrimination of five urinary tract infection pathogens cultured on blood agar plates based on hyperspectral techniques. William R. Windham et al. [12,13] explored the feasibility of the classification of six non-O157 *Escherichia coli* (O26, O45, O103, O111, O121 and O145) cultured on rainbow agar media. Simone Arrigoni et al. [14] studied the classification of *Escherichia coli*, *Enterococcus faecalis*, *Staphylococcus aureus*, *Proteus mirabilis*, *Proteus vulgaris*, *Klebsiella pneumoniae*, *Pseudomonas aeruginosa* and *Streptococcus agalactiae* cultured on sheep blood agar and achieved good classification rates of 99.5%. Kammies et al. [15] investigated the potential for near-infrared (NIR) hyperspectral imaging to be used to distinguish *Bacillus cereus, Escherichia coli, Salmonella enteritidis, Staphylococcus aureus* and *Staphylococcus epidermidis* grown on Luria–Bertani (LB) agar and the best predictions were made for *B. cereus* and *Staphylococcus* species, where results ranged from 82.0% to 99.96% correctly predicted pixels. Arne Walter et al. [16] achieved the distinction of *Bacillus thuringiensis*, *Escherichia coli*, *Bacillus atrophaeus* and *Bacillus subtilis* by method of bacteria spectra based on hyperspectral laser induced fluorescence. However, it only explained the distinction of bacteria from the direction of bacteria spectra instead of statistics. Alberto Signoroni et al. [17] combined hyperspectral images and convolutional neural networks (CNN) to achieve the identification of nine kinds of urinary tract infection species cultured on 5% sheep blood agar plates and acquired the best classification accuracy of 99.7%. Feng et al. [18] used hyperspectral technology to classify three strains of *Escherichia coli* including *E. coil* O8, O11 and O138, two strains of *Listeria* including *L. monocytogens* and *L. seeligeri* and *Staphylococcus aureus* cultured on tryptone soybean agar (TSA) medium and it showed the best overall classification accuracy of 96%. However, bacterial detection based on hyperspectral imaging is greatly affected by the media. They only studied the classification of bacteria in either one common bacterial culture environment or selective medium. In case of the absence of one common agar medium in the laboratory, bacterial detection could not be achieved successfully. To establish a more adaptable model and predict the bacterial species without restriction of any medium, this paper proposes the classification of bacterial colonies on different agar media based on hyperspectral imaging. In detail, the paper was committed to achieve the classification of three kinds of bacteria, *Escherichia coli*, *Staphylococcus aureus* and *Salmonella*, cultured on three kinds of agar media, Luria–Bertani agar (LA), plate count agar (PA) and tryptone soy agar (TSA).

## 2. Results and Discussion

### 2.1. Spectral Analysis of Bacterial Colonies

**Figure 1:**
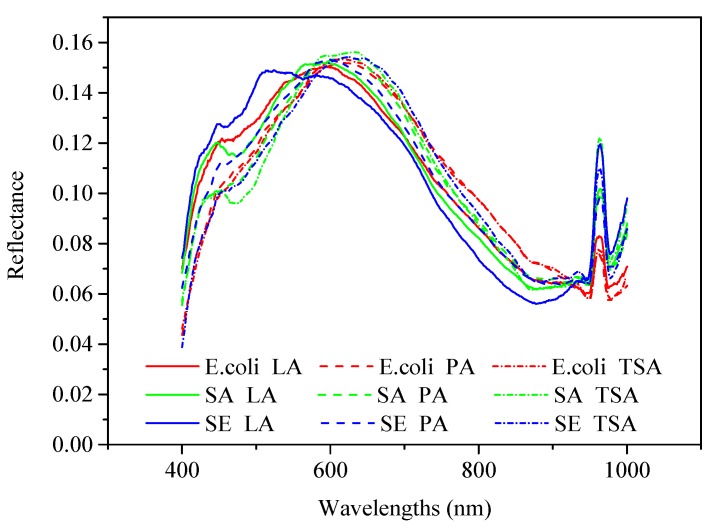
Spectra of bacterial colonies. Note: E. coli, SA and SE are abbreviations of *Escherichia coli*, *Staphylococcus aureus* and *Salmonella*, respectively; LA, PA and TSA are abbreviations of Luria–Bertani agar, plate counting agar and tryptone soy agar, respectively. For example, E. coli LA represents the *Escherichia coli* cultured on Luria–Bertani agar.

The spectra acquired not only contained the spectral information of the bacteria, but also those of the medium. Figure 1 shows the reflectance spectra of three foodborne pathogens cultured on three media. From the view of the culture medium (the dotted lines and lines of the same color in Figure 1), the different culture media have great influence on the spectrum of the bacterial colonies which increases the difficulty of classification. In the range of 400–580 nm, the spectral reflectance of the same bacterial colonies cultured on TSA medium is the smallest compared to the other two media, and the spectral reflectance of the same bacterial colonies cultured on LA medium is the largest compared to the other two media. Interestingly, in the 580–900 nm range, an opposite trend was observed where the spectral reflectance of the same bacteria colonies cultured on the LA medium becomes smallest compared to the other two media. Regarding bacterial strains (the solid or dotted lines of different colors in Figure 1), the regions with significant changes in the spectrum are concentrated in the range of 400–580 nm. The peak of *Staphylococcus aureus* at 450 nm is more obvious than that of either *Salmonella* or *Escherichia coli* and all three bacteria have obvious peaks at 960 nm. 

### 2.2. Principal Component Analysis

**Figure 2:**
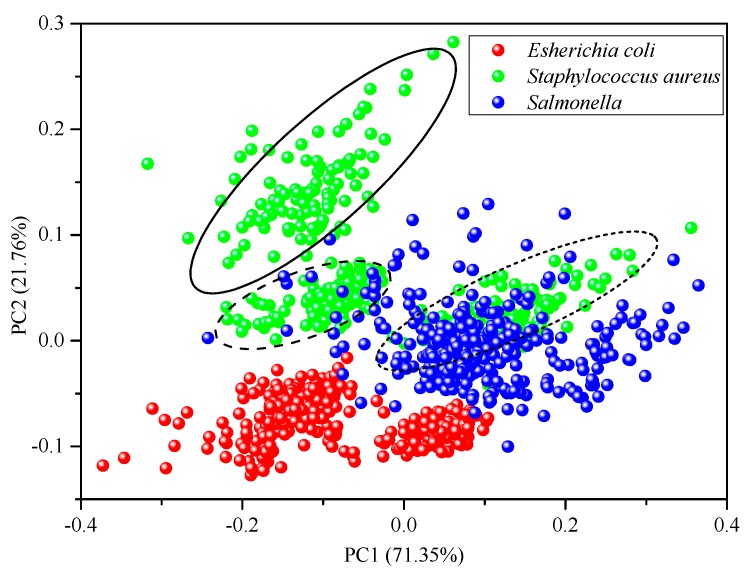
Score plot of bacterial colonies.

Principal component analysis (PCA) was performed on the spectral data of the calibration set samples pretreated by MSC in the full spectral range to show the distribution samples due to the effect of different culture agars. The variance contribution rates of the first two principal components (PCs) were 71.35% and 21.76%, respectively, resulting in a cumulative contribution rate of 93.11%. This means that the first two PCs could basically explain the majority of variance of the original spectral data. A two-dimensional scatter plot based on PC1 and PC2 is shown in Figure 2, where the red, green and blue markers represent *Escherichia coli*, *Staphylococcus aureus* and *Salmonella* bacteria respectively. There is a trend for the separation of the three bacteria with *Escherichia coli* samples well isolated from the other two bacteria and *Salmonella* samples significantly overlapping with part of the *Staphylococcus aureus* samples. Interestingly, it was observed that each of the bacterial species tended to form into three clusters. Such grouping turned out to be closely related with the types of agars. For instance, the three clusters indicated by the ellipse, dashed ellipse and the dotted ellipse are actually *Staphylococcus aureus* samples cultured on TSA, PCA and LA, respectively. This clearly demonstrated that great variations could be introduced into the spectral profiles of bacterial colonies if different agars are used as culture medium. In other words, using different agars can propose great difficulties for bacterial classification since it extends the distribution space of the same bacterial species so that bacterial colonies from different species are more prone to overlapping. Nevertheless, the two-dimensional scatter plot based on PC1 and PC2 could roughly distinguish the range of bacterial distribution in the sample. It showed that it was potentially possible to classify *Escherichia coli*, *Staphylococcus aureus* and *Salmonella* under the background of TSA, PA and LA medium. However, a supervised pattern recognition method is needed for further classification.

### 2.3. Full Wavelength Models

PLS-DA and SVM were employed to establish the full wavelength classification models. Table 1 shows the classification performance of the full-wavelength model and Table 2 shows the confusion matrix of prediction for the linear PLS-DA and non-linear GOA-SVM classification models.

#### 2.3.1. PLS-DA Classification Models

In the establishment of PLS-DA full wavelength models, a full cross-validation method was employed to select the number of latent variables (LVs). Table 1 shows that by using five latent variables, the OCCRs of calibration and prediction and the Kappa coefficient are 75%, 74.03% and 0.71, respectively. The results for prediction showed that only 80.39% of *E. coli* samples, 75.59% of *S. aureus* samples and 61.13% of *Salmonella* samples were correctly classified. Under further investigation, it was found that 7% of *E. coli* samples were misclassified as *S. aureus* and 9.51% and 14.71% of *S. aureus* samples were misclassified as *E. coli* and *Salmonella*, respectively. Moreover, 38.24% of *Salmonella* samples were misclassified as *S. aureus* and 12.6% of *E. coli* samples, 0.196% of *S. aureus* samples and 0.63% of *Salmonella* samples were not identified as any of the three bacteria. It was indicated that direct classification of bacterial strains under the background of three media by the partial least squares discriminant analysis (PLS-DA) linear method is not satisfactory. This might have been due to the overwhelming dominance of agar variations in the spectra over those by bacterial colonies.

#### 2.3.2. SVM Model Optimization

The nonlinear method SVM was employed to project spectral information into high-dimensional space to highlight the spectral characteristics of bacteria in order to achieve better classification of bacterial species. In SVM modeling, GA, PSO and a recently developed algorithm named GOA were employed to optimize the two important parameters, i.e., the penalty coefficient c_p_ of the SVM and the RBF kernel function parameter g. As can be seen in Table 1, all three kinds of SVM models showed good performance in the classification of bacterial colonies. The OCCRs for calibration set and prediction set and Kappa coefficient of PSO-SVM model were 99.45%, 98.78% and 0.98, respectively. Although the OCCR for calibration set of PSO-SVM model was slightly smaller than that of the GA-SVM model, PSO-SVM outperformed GA-SVM in terms of OCCR Kappa coefficient. Nevertheless, GOA-SVM with OCCRs of 99.45% and 98.82% for calibration set and prediction set and Kappa coefficient of 0.98 showed the best performance for bacterial colony classification in this study. Among the predicted results of GOA-SVM, 97.33% of *E. coli* samples, 99.71% of *S. aureus* samples and 99.16% of *Salmonella* samples were correctly classified. For the wrong classifications, 2.67% of *E. coli* samples and 0.29% of *S. aureus* samples were misjudged as *Salmonella*, 0.63% of *Salmonella* samples were misjudged as *E. coli* and 0.21% of *Salmonella* samples were misjudged as *S. aureus* with no unclassified samples. Compared with the PLS-DA model, the OCCRs and Kappa coefficient of the optimal SVM model were much higher and all samples were assigned to a certain class category. Compared with the traditional GA-SVM and PSO-SVM models, the GOA-SVM full wavelength model had better classification performance in this study. Therefore, GOA-SVM full-wavelength model was the best tool for classifying bacterial species under the interference of three background media used in this study.

### 2.4. Simplified Classification Models

In this study, three wavelength selection methods, including competitive adaptive reweighted sampling (CARS), successive projections algorithm (SPA) and genetic algorithm (GA) were employed to allocate important wavelengths that can be used to simplify the classification models established by applying PLS-DA and GOA-SVM. The performance of the model is shown in Table 3.

It was demonstrated that the PLS-DA model simplified by CARS, SPA and GA remained unsatisfactory since the OCCRs for calibration set and prediction set were both lower than 80%, and the Kappa coefficient less than 0.65. Therefore, it was still difficult to classify bacterial species directly using the original spectra where the information of different media was involved. For the simplified models based on nonlinear methods, the CARS-GOA-SVM simplified model showed the best performance in all simplified models. The best OCCR for calibration set of CARS-GOA-SVM model was 99.45%, which was the same as GA-GOA-SVM model and slightly higher than that of the SPA-GOA-SVM model with an OCCR of 99.24% for calibration set. In terms of prediction, CARS-GOA-SVM model produced an OCCR of 98.73% and a Kappa coefficient of 0.98, which outperformed both SPA-GOA-SVM model and GA-GOA-SVM model where OCCRs and Kappa coefficient of 98.69%, 0.98 and 98.60%, 0.98 were attained.

Moreover, the CARS-GOA-SVM simplified model performed equivalently to the full-wavelength SVM model with similar OCCRs and Kappa coefficient. However, considering the time and efficiency during model establishment, CARS-GOA-SVM was the best choice for classifying bacteria without removing any medium of three kinds of media.

**Figure 3:**
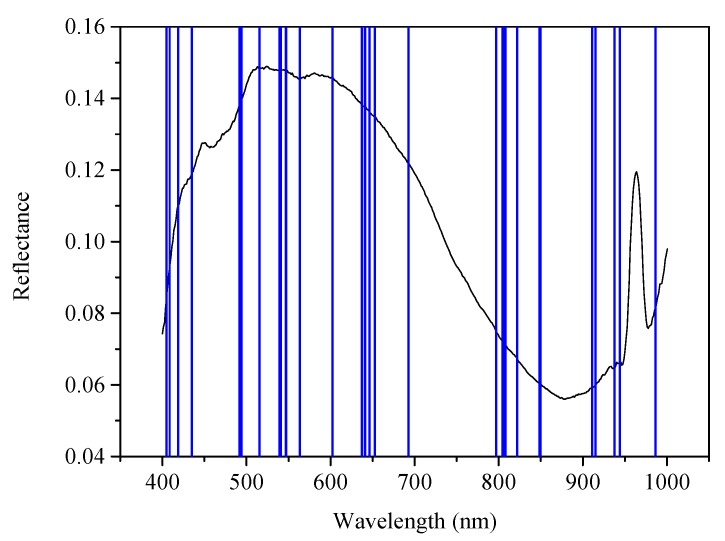
Wavelength selected by competitive adaptive reweighted sampling (CARS).

Therefore, the optimal model for classifying bacterial species in the context of different agar interference was GOA-SVM model based on wavelengths selected by CARS. In this study, 30 characteristic wavelengths were selected by CARS, as shown in Figure 3. The confusion matrix of prediction set of CARS-GOA-SVM simplified model is shown in Table 4. It was shown that 97.48% of *E. coli* samples, 99.80% of *S. aureus* samples and 98.32% of *Salmonella* samples were correctly predicted. Only 2.52% of *E. coli* samples and 0.2% of *S. aureus* samples were misjudged as *Salmonella*; 1.47% of *Salmonella* samples were misjudged as *E. coli*; and 0.21% of *Salmonella* sample was misjudged as *Staphylococcus aureus*. 

### 2.5. Pixel Analysis

To further check the performance of the established classification model, pixel-level predictions were evaluated [19]. The GOA-SVM full wavelength model and the CARS-GOA-SVM simplified model previously established based on colony-level spectral data in the calibration set were applied to each pixel of spectral images for the prediction set to predict the pixel-wise bacterial labels. The performance of predicting pixel-level spectral data using existing GOA-SVM and CARS-GOA-SVM models is shown in Table 5. The pixel-level OCCR for prediction of the existing CARS-GOA-SVM model was 93.32%, which was higher than existing GOA-SVM full wavelength model with an OCCR of 91.86%. The correct discrimination rates of *E. coli*, *Staphylococcus aureus* and *Salmonella* for the CARS-GOA-SVM model were 95.35% (46712/48989), 95.51% (14138/14802) and 80.09% (7981/9965), respectively, which were higher than that of GOA-SVM model of 94.99% (46531/48989), 90.77% (13436/14802) and 78.06% (7779/9965), respectively. Interestingly, the OCCR of the CARS-GOA-SVM in predicting *Salmonella* in colony-level under different background was 98.32%, but only 80.09% was achieved for the pixel-level prediction. This may be due to the fact that some non-bacterial pixels were used as bacterial spectra when determining the region of interest for *Salmonella* but these effects could be submerged due to the average operation. The above results showed that the model established by colony-level spectral data could also be used to predict pixel-level spectral data, though the accuracy should be further improved.

## 3. Materials and Methods 

### 3.1. Bacterial Culture and Sample Preparation

Frozen stored *Escherichia coli*, *Staphylococcus aureus* and *Salmonella* (−80 °C) were streaked into tryptone soy agar (TSA) and activated in a constant temperature incubator at 37 °C for 20 ± 2 h. A single colony with good morphology was inoculated into Luria–Bertani (LB) and cultured for 4h in a shaker at 37 °C. Then the resultant bacterial solution was diluted to a concentration of about 10^3^ CFU/mL using phosphate buffered saline (PBS). 100 µL aliquot of bacterial suspension was inoculated in TSA, PA and LA by the spread plate method. The plate was inverted and placed in a 37 °C constant temperature incubator for 20 ± 2 hours. The above experiment was repeated for another day where the bacterial concentration dilution ranged 10^2^–10^4^ CFU/ml. The data attained for the first day was used for calibration and the second day for validation. 

The food-borne pathogens (*E. coli*, *Staphylococcus aureus* and *Salmonella*) and phosphate buffered saline (PBS) required for the experiment were provided by the Biological Laboratory of Animal Science School of Huazhong Agricultural University, Wuhan, China. Tryptone soy agar, plate count agar, Luria–Bertani agar and coating stick were all provided by Qingdao Haibo Biotechnology Co., Ltd, Qingdao, China. The experimental water was double distilled water.

### 3.2. Hyperspectral Image Acquisition

**Figure 4:**
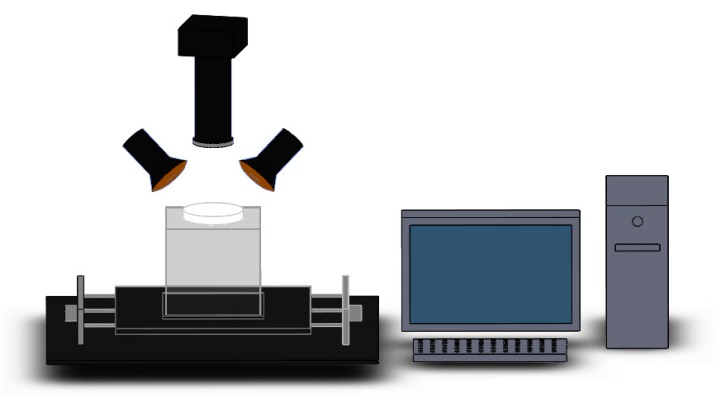
Hyperspectral imaging system.

A hyperspectral imaging system (Zolix, Beijing, China) as described elsewhere [18] was used in the experiment. As shown in Figure 4, the system consists of a hyperspectral camera (V10E-CL, Specim, Oulu, Finland), halogen light source (OSRAM, DECOSTAR51, MR16, Berlin, Germany), a personal computer, a high-precision electronically controlled translation stage (Zolix, China). A homemade acrylic transparent sample holder sized 210 mm × 210 mm × 130 mm was designed to eliminate background interference. Hyperspectral images were recorded in the wavelength range of 400∼1000 nm with a spectral resolution of 2.8 nm and a spectral interval of 1.25 nm. To avoid distortion in the hyperspectral images, the exposure time of the camera was set as 100 ms and the platform moved at a speed of 1.6 mm/s. The bottom of the sample holder was a blackboard with reflectivity close to zero. The Petri dish was placed in the hollow position of the acrylic transparent sample holder. The distance between the Petri dish and the lens of the camera was about 27 cm.

In order to reduce the influence of camera dark current and external noise interference in image acquisition, the following equation was employed to calibrate the original image.

(1) $$R=\frac{I-D}{W-D}$$

where *R* is the reflectance image after calibration; *I* is the original hyperspectral reflectance image; *W* is a standard white board image; *D* is a full black background image acquired by covering camera lenses while turning off the light source.

### 3.3. Hyperspectral Image Processing and Data Acquisition

**Figure 5:**
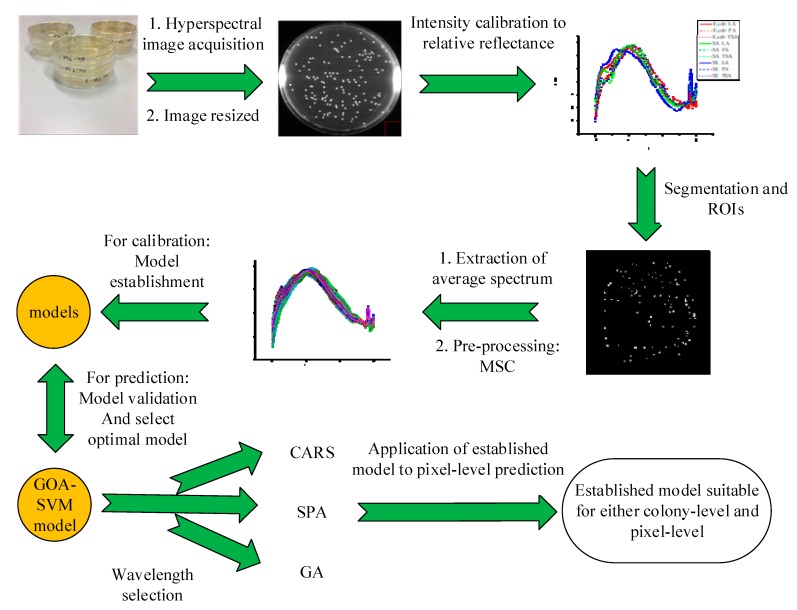
Flowchart of spectral data acquisition and analysis.

The hyperspectral image acquisition and processing is shown in Figure 5. The corrected image was resized into three-dimensional images to contain 370 × 370 × 520 pixels. Due to the low signal to noise ratio of the image obtained at the two ends of the investigated wavelength range, images at the first seven bands and the last thirteen bands were eliminated resulting in a total of 479 images for the final hyperspectral data cube. 

In order to extract the spectral information of the target area, after scrutinizing the spectral difference between the bacterial colonies and the background agar, the band image at 672 nm showing large contrast was selected for further image segmentation. Based on this band image, a histogram was established and the gray threshold for segmenting bacterial colony from background agar was determined at the maximum gray frequency in the gray histogram. The threshold varied from image to image. After segmentation, a binary image named as mask was obtained, which was further improved by removing noise and specular reflection area. The noise was removed by using morphological processing of image open and the spectral reflection area was eliminated by removing the two largest eight-connected regions in the primary mask. The average spectrum of bacterial colonies in the corrected image was extracted from the identified region of bacterial colonies in the mask image and used as the original spectrum of the sample. To reduce the noise signal and enhance the spectral information of bacteria, multiplicative scatter correction (MSC) was employed to process the spectra.

### 3.4. Sample Set Establishment

As shown in Table 6, 916 samples were collected for calibration, including 103, 83 and 94 samples of *Escherichia coli* 110, 114 and 101 samples of *Staphylococcus aureus* and 96, 108, 107 samples of *Salmonella* cultured on LA, PCA and TSA media, respectively. A total of 2210 samples were collected for prediction, including 325, 167 and 222 samples of *Escherichia coli*, 347, 289 and 384 samples of *Staphylococcus aureus* and 114, 211, 151 samples of *Salmonella* cultured on LA, PCA and TSA media, respectively. 

### 3.5. Model Establishment and Optimization

Principal component analysis (PCA) [20] is a dimensionality reduction statistical method that can transform the original data into a new set of irrelevant variables by orthogonal transformation and extract certain integrated variables to explain the original data information. Therefore, before model development, PCA was utilized to visualize the clustering of bacterial colonies in the context of different culture agars. For model establishment, Partial least squares discriminant analysis (PLS-DA) and support vector machine (SVM) were employed.

Partial least squares discriminant analysis (PLS-DA) is a linear discriminant analysis method based on partial least squares regression (PLSR). *Escherichia coli*, *Staphylococcus aureus* and *Salmonella* colonies were classified as Category 1, 2 and 3, respectively. The PLS regression model based on spectral data X and class label Y was established by using the calibration set samples, and prediction set samples was predicted by this model, with 0.5 as the threshold. If the difference between the predicted value and the actual label value of the sample was within ±0.5, the sample was correctly classified; otherwise a wrong classification was made. 

Support Vector Machine (SVM) is a learning machine based on statistical learning theory, which can better solve the classification problem of non-linear and high-dimensional data and is widely used in the field of hyperspectral imaging. The basic idea of SVM is to find hyperplanes that can distinguish different samples and make the hyperplane farthest from the samples of different groups [21]. Traditional optimization algorithms (including genetic algorithm (GA) [22], and particle swarm optimization (PSO) [23]) and a new optimization algorithm named grasshopper optimization algorithm (GOA) [24] were used to optimize the penalty coefficient c_p_ and RBF kernel function parameter g of SVM to make the classification accuracy of the classification model optimal and stable.

### 3.6. Grasshopper Optimization Algorithm (GOA)

The grasshopper optimization algorithm was first proposed by Seyedali Mirjalili et al. in 2017 by imitating the food-seeking behavior of a grasshopper population in nature [25]. The grasshopper population forms a network, which connects all grasshopper individuals and coordinates the positions of each individual. The individual can decide the direction of preying through other individuals in the group. It has good convergence ability toward the optima. In general, GOA advances itself strongly to exploitation [26].

The grasshopper algorithm firstly needs to create a search space and the location of the grasshopper, which is randomly distributed. Then, according to Equation the position of individuals in grasshopper population is updated.

(2) $$X_{i}^{d}=c\left(\overset{N}{\underset{i=1,\;i\neq j}{\sum }}c\frac{ub_{d}-lb_{d}}{2}\;s\left(\left|x_{j}^{d}-x_{i}^{d}\right|\frac{x_{j}-x_{i}}{d_{ij}}\right)+{\hat{T}}_{d}\right)$$

where $ub_{d}$ is the upper limit of the d-th dimension; $lb_{d}$ is the lower limit of the d-th dimension; ${\hat{T}}_{d}$ is the target location of the optimal solution of the *d*-th dimension so far in the search scope; $\left|x_{j}^{d}-x_{i}^{d}\right|$ is the distance between the *i*-th and *j*-th grasshoppers in the *d*-th dimension; $\frac{x_{j}-x_{i}}{d_{ij}}$ is the unit vector from the *i*-th to the *j*-th grasshopper; *s* is the main component of social interaction, which defines the direction of grasshopper movement; *c* is the decreasing coefficient of comfort zone, exclusion zone and attraction zone of grasshopper and is the main control parameter of GOA optimization algorithm. The definitions of s and c are shown in Equations (3) and (4).

(3) $$s\left(r\right)=fe^{-\frac{r}{l}}-e^{-r}$$

(4) $$c=c_{max}-l_{iter}\frac{c_{max}-c_{min}}{L}$$

where f indicates the intensity of attraction, l is the attractive length scale, L is the maximum number of iteration, $l_{iter}$ is the current iteration, and $c_{max}$ = 1, $c_{min}$ = 0.00004 in this study. 

The value of the first two dimensions of the result of each iteration (the position of grasshoppers) is used as the penalty coefficient c_p_ and RBF kernel function parameter g of SVM to calculate the fitness value, and the fitness value is compared for each iteration. The maximum fitness value in each iteration is found and the global maximum is taken when the iteration is completed. The fitness function is defined as follows:

(5) $$\mathrm{Fitness}=\frac{1}{K}\overset{K}{\underset{k=1}{\sum }}\frac{1}{N}\overset{N}{\underset{j=1}{\sum }}\delta \left(y_{pre},y_{true}\right)$$

where K is the cross-validation number, N is the number of calibration sets and δ is the relation between $y_{pre}$ and $y_{true}.$ That is, if $y_{pre}$ = $y_{true}$, δ = 1; otherwise δ = 0.

### 3.7. Wavelength Selection

Spectral variables (479 in total) in the full-wavelength model were analyzed, which caused a huge amount of calculation. Therefore, establishing a simplified model without reducing the spectral information of bacteria is necessary. To achieve it, three wavelength selection methods, including competitive adaptive reweighted sampling (CARS), successive projections algorithm (SPA) and genetic algorithm (GA) were employed.

### 3.8. Model Assessment

The performance of the model is determined by the overall correct classification rate (OCCR), confusion matrix and Kappa coefficient [27,28]. OCCR is the ratio of the correct number of samples predicted by the model to the total number of samples. The formula is as follows:

(6) $$\mathrm{OCCR}=\frac{Correct\;number\;ofsamples}{Total\;number\;of\;samples}\times 100\%$$

The Kappa coefficient is a method for assessing consistency in statistics. It can be used as an evaluation criterion for the accuracy of multi-class models. The value range is generally [0,1]. The higher the Kappa coefficient value, the higher the classification accuracy of models is. The formula is as follows:

(7) $$K=\frac{P_{all}-P_{e}}{1-P_{e}}$$

where $P_{all}$ is the total classification accuracy, $P_{e}$ is expressed as $P_{e}=\frac{\sum_{i=1}^{c}a_{i}\times b_{i}}{N\times N}$, where $a_{i}$ is the number of samples of the i-th real category, $b_{i}$ is the number of samples of the i-th predicted category and N is the total number of samples. 

## 4. Conclusions

Bacterial characterization based on hyperspectral imaging has been intensively studied, but by only considering one bacterial culture medium. Detection of bacteria on different types of agars will provide a unified and much convenient way in classifying bacteria. Taking three bacterial species of *Escherichia coli*, *Staphylococcus aureus* and *Salmonella* cultured on LA, PA, TSA as examples, this study developed a simpler and more universal method for classification of bacterial species based on hyperspectral imaging by considering the effect of medium spectra. The spectral data of bacterial colonies including the spectral information of bacteria and culture medium were extracted from the region of interest, and a linear PLS-DA and non-linear SVM full-wavelength classification model and simplified model were established. It was found that OCCRs for calibration and prediction were both less than 75% and the Kappa coefficient less than 0.71 for the PLS-DA model which demonstrated its deficiency in classifying bacterial strains under the background of three media. The full-wavelength SVM model showed great results despite the influence of LA, PA and TSA on spectra and the best performance was attained by the GOA-SVM model whose OCCRs for calibration and prediction and Kappa coefficient were 99.45%, 98.82% and 0.98, respectively. Moreover, in order to make the model predict performance with higher precision and lower computational load, PLS-DA and GOA-SVM simplified models were established by employing different wavelength selection method including CARS, SPA and GA. The CARS-GOA-SVM turned out to be the best model for classifying bacteria in this study. In addition, pixel analysis was carried out and it was shown that pixel-level data could also be employed for bacterial classification, although the performance was not as good as that of colony-level data. Therefore, foodborne pathogenic bacteria colonies can be well classified using a hyperspectral technique and machine learning methods despite the presence of spectral interference from Luria–Bertani agar (LA), plate count agar (PA) and tryptone soy agar (TSA). Moreover, the application of GOA was more powerful than traditional algorithms in optimizing model performance and thus could be investigated for more applications. Nevertheless, future work can be conducted to investigate the spectral features of colonies that contribute to high specificity and sensitivity of the classification models.

## Figures and Tables

**Table 1:** Performance of full wavelength models.

Model	Optimization Methods	LVs	c_p_	g	OCCR (%)		Kappa
					Calibration	Prediction	
PLS-DA	-	5	-	-	75	74.03	0.71
SVM	GA	-	41.74	51.53	100	97.33	0.96
	PSO	-	13.69	16.56	99.45	98.78	0.98
	GOA	-	70.61	5.49	99.45	98.82	0.98

**Table 2:** Confusion matrix of prediction for full-wavelength models.

			Predicted Label			
		Bacteria	E. coli	SA	SE	Unclassified
PLS-DA	True Label	E. coli	80.39%	7%	0	4.30%
		SA	9.51%	75.59%	14.71%	
		SE	0	38.24%	61.13%	
GOA-SVM	True Label	E. coli	97.33%	0	2.67%	0
		SA	0	99.71%	0.29%	
		SE	0.63%	0.21%	99.16%	

Note: E. coli, SA and SE are abbreviations of *Escherichia coli*, *Staphylococcus aureus* and *Salmonella*, respectively.

**Table 3:** Performance of PLS-DA and GOA-SVM simplified models.

Wavelength Selection Methods	Number of Wavelengths	PLS-DA			GOA-SVM		
		OCCR_C_%	OCCR_P_%	Kappa	OCCR_C_%	OCCR_P_%	Kappa
CARS	30	79.15	71.72	0.63	99.45	98.73	0.98
SPA	24	75.66	71.72	0.62	99.24	98.69	0.98
GA	69	78.50	73.53	0.64	99.45	98.60	0.98

Note: PLS-DA: partial least squares discrimination analysis; GOA-SVM: grasshopper optimization algorithm support vector machine.

**Table 4:** Confusion matrix of training set for CARS-GOA-SVM simplified model.

	Bacteria	Predicted Label			Unclassified
		*E. coli*	SA	SE	
True Label	E. coli	97.48%	0	2.52%	0
	SA	0	99.8%	0.20%	
	SE	1.47%	0.21%	98.32%	

Note: E. coli, SA and SE are abbreviations of *Escherichia coli*, *Staphylococcus aureus* and *Salmonella*, respectively.

**Table 5:** Comparison of performance of predicting pixel-level spectral data using existing GOA-SVM and CARS-GOA-SVM models.

			Predicted Label			
		Bacteria	*E. coli*	SA	SE	OCCR_P_ (%)
GOA-SVM	True Label	*E. coli*	94.99%	0.12%	4.89%	91.86
		SA	6.09%	90.77%	3.14%	
		SE	21.06%	0.88%	78.06%	
CARS-GOA-SVM	True Label	*E. coli*	95.35%	0.12%	4.53%	93.32
		SA	1.18%	95.51%	3.31%	
		SE	19.27%	0.64%	80.09%	

Note: E. coli, SA and SE are abbreviations of *Escherichia coli*, *Staphylococcus aureus* and *Salmonella*, respectively.

**Table 6:** Number of samples in the calibration and prediction set.

	Calibration Set				Prediction Set			
	LA	PA	TSA	Total	LA	PA	TSA	Total
E. coli	103	83	94	280	325	167	222	714
SA	110	114	101	325	347	289	384	1020
SE	96	108	107	311	114	211	151	476
Total	309	305	302	916	786	667	757	2210

Note: E. coli, SA and SE are abbreviations of *Escherichia coli*, *Staphylococcus aureus* and *Salmonella*, respectively; LA, PA and TSA are abbreviations of Luria–Bertani agar, plate counting agar and tryptone soy agar, respectively.
